# Treatment of Gunshot Wounds

**Published:** 1894-04

**Authors:** Willis F. Westmoreland

**Affiliations:** Atlanta, Ga.


### TREATMENT OF GUNSHOT WOUNDS.*

By WILLIS F. WESTMORELAND, M.D.,
ATLANTA, GA.

The object of this paper is more to learn something by the discussion which it may bring out than what I may be able to say myself. There are some points in gunshot wounds on which I am in doubt.

The first of this class of injuries that I was called upon to treat was shortly after I graduated, and it thoroughly convinced me of

*Read at the meeting of the Southern Surgical and Gynecological Association, New Orleans, November, 1893.

the fallacy of probing. I saw the case with a fellow-practitioner of age and experience. The patient was seen within an hour after the injury, which was self-inflicted with suicidal intent; the bullet entered near the right nipple. When we saw him there was no constitutional disturbance, nor was there up to the time we discharged him as cured, about a week afterward. We made a most thorough local examination, and probed to the complete satisfaction both of ourselves and a group of friends and relatives who watched us at first with a silent but critical interest, which finally gave way to admiring murmurs of satisfaction as they watched the carefulness of our search. As the result of our examination, we decided most positively that the bullet had not entered the thoracic cavity, but was lodged somewhere in the pectoral muscles. This patient died some six weeks afterwards, and the physician who made the *post mortem* told me that he found the right pleural cavity full of pus, and traced the bullet through the lung, diaphragm and liver into the right kidney, where he found it encysted.

A retrospective view of the symptoms which developed in this case convinces me that had we not formed such a positive opinion as to the position of this ball we would have associated his remote symptoms with the injury and arrived at a diagnosis in time to have operated and saved the patient's life. This case furnished much food for reflection

So far as gunshot wounds are concerned, early operation gives good results.

In the discussion of gunshot wounds it is better to begin with the projectile that inflicts the injury. The improved arms of today, discharging a conical bullet from a highly charged cartridge, through a rifled barrel, has a penetrating power unknown and unappreciated in former writings upon this subject. It inflicts a wound more on the order of a punctured than the former lacerated contused wounds, and there is less comminution when a bone is struck; nor is deflection so apt to occur. The wound of exit is also more apt to correspond to that of entrance, and there is less danger of infection from the cartridge ball than from the old powder and ball weapon, which was also liable to carry the wadding into a wound. There is no longer any mystery about gunshot wounds, though there are erroneous teachings. Particularly is this true in regard to the bullet generating enough heat in its passage to destroy germs that may have lodged on it. Even so prominent a writer as Roswell Park, in an article on the early treatment of gunshot wounds, claims for the bullet that "it is so heated in its passage out of the weapon and through the air that it kills any germs which may have found lodgment on it, *i. e.*, it enters the body in an aseptic condition." The experiments of La Garde (Louis A., *New York Medical Journal*, October 22, 1892) controvert this, and these experiments conclusively prove that "the heat developed by the act of firing is not sufficient to destroy all the organic matter on a projectile."

When a bullet enters the human body and penetrates a cavity, and particularly that of the abdomen, its location is a simple unknown quantity.

*Shock.*—Shock is out of proportion to the injury in a great many cases of civil practice; and this is likewise true in army practice. A man may have a limb carried away without directing his attention to it. Of course, that is primary shock. In shock of this kind the usual remedies are given, and in a short time the surgeon can tell from the constitutional symptoms as to whether the injury is one of a serious character.

In regard to cerebral injuries, I think the profession is in a rather mixed condition—that is, in cases where we have fractures the result of gunshot wounds. I am thoroughly convinced in my own mind that all cases with symptoms of depressed fracture should be relieved. As to interference with the brain, I am in some doubt whether we should probe or not. You can follow a bullet into the brain with an elastic catheter without doing much damage to the integrity of the cerebral substance. With a stiff probe introduced with the idea of following the bullet, you probe down through a sinus or a septum between the cerebral tissue in different portions of the brain other than that in which the bullet was discovered *post mortem;* in other words, the probing has a good deal to do with killing the patient. The bullet is found perhaps an inch from the point of entrance, exploration never having revealed it at all. Another point in regard to cases of brain surgery from gunshot wounds is the fact that it may be taken for granted that you have removed all sources of irritation that have produced the epilepsy. As a sequel of gunshot wounds you may have either infection, hemorrhage, abscess, or hernia. I am satisfied that the majority of cases of hernia that result fatally are due to cerebral abscess. As early as 1840, Gordon Buck (*New York Medical Journal*), in his report of thirty-three cases, states that in all in which he had an opportunity of making an examination, an abscess was found in the substance of the brain or upon its surface in the immediate vicinity of the hernia.

If you open the abscess there is no reason why you should not get perfect results, and the history of surgery does not show a recovery from cerebral abscess without operative interference; that is, your patient never gets well, and only operative interference will cure.

In regard to gunshot wounds of the lungs we never probe them. There is no necessity of seeing whether the bullet goes into the cavity or not. The constitutional symptoms will reveal the fact that the shock caused by the emotion of the patient has disappeared; the character of the pulse and temperature will reveal to you whether it has penetrated the cavity or not.

Death from gunshot wounds of the lung must occur from two causes, either hemorrhage or infection. No large vessel can be severed at the extreme lower portion. It is possible to have infection from the bronchi (large); the smaller bronchi and air vesicles are particularly aseptic as a rule; at least 75 per cent. of gunshot wounds are aseptic.

Probing is an additional source of danger. It, like the removal of the bullet, tends to displace the clots and unseal vessels, even if infection is not carried into the wound in this way. There is little deflection by soft tissues with our improved arms, except in cases of tendons and fascia.

The use of a high degree of heat and chemical agents render the projectiles inflicting them sterile or free from septic germs, and we can even go further and say that though the projectiles become infected from careless handling, it is possible, but not probable, that even an infected bullet will not infect a properly treated wound. I have never seen pus in a wound that was promptly occluded, without probing or other meddlesome interference, and I think I am perfectly safe in saying that 90 per cent. of properly treated wounds (gunshot) are aseptic. These wounds always bleed sufficiently for nature to form an occlusive blood-clot. An antiseptic cleansing of the surrounding parts, a piece of protective or rubber tissue to insure the filling of the wound with blood, and the application of an antiseptic dressing, with perfect rest of the part, will insure the perfect organization of the part as we have after operations—that is, union by the moist blood-clot.

*Probe.*—I never use a probe in a gunshot wound except as it may become necessary in the progress of a *formal antiseptic operation.* The probing rarely does any good beyond satisfying a morbid curiosity. Even if your wound is not infected by the probe itself, it allows the entrance of air. It destroys nature's occlusive blood-clot, and in this way prevents prompt union. Shreds of clothing are easily discovered, and if the bullet is, its removal is generally contraindicated, for if discovered near the surface in another part, its removal at this time simply affords, through the two openings, double opportunities for infection. The probing may start a troublesome hemorrhage which had ceased. A bullet imbedded in the tissues is an inoffensive substance.

A gunshot wound of the abdomen, if it is penetrating, will nearly always prove fatal without operative interference. The best statistics place the mortality at 91 per cent. In armies they do not count patients who are wounded, but cases which have recovered from primary shock and get into the hands of the surgeon. Both German, French, and American statistics of the last rebellion give over 91 per cent.; but we have got to take statistics in inverse ratio. The question is not how they die from operative interference, but how many patients can we save by it? The mortality statistics being over 91 per cent. without interference, how can we reduce them to the 80's? I am confident that a great many of these patients can be saved by early operative interference. In perforating gunshot wounds of the intestines patients die promptly in twenty-four, thirty-six, or forty-eight hours.

I do not propose to give the method of operating in these cases, for any one who does surgery knows the indications will be pointed out after the abdomen is opened.

There is one point on which I desire to express an opinion. The physician calls in the abdominal surgeon, and although he expresses himself very radically about operations involving the removal of the ovaries and tubes, etc., when he comes to these cases it is opium and wait. When he comes to the bedside and sees the case before him, he does not have the courage of the conviction he has so openly avowed. Particularly is this true of those gynecologists and cœliotomists who, like toadstools, spring up in a night. Such men are called in for support, but we do not get it. An early operation in these cases is imperative, particularly so where you have profound shock which indicates perforation of the bowel, or where you have hemorrhage. In such cases your patient will die in twenty-four or thirty-six hours after operation. The fact that the bowel has not been perforated, or that you do not have hemorrhage, is no indication that the patient will not die. Statistics show conclusively that patients with multiple penetrating wounds of the abdomen, which have not made perforations of the bowel—you may call them simply wounds of the peritoneum—will die. These cases mislead you. You have no profound shock, the patient rallies from it, goes to bed, and later dies. Examination shows septic peritonitis. After the third day you could not operate successfully, because you have a subnormal temperature. After the temperature has descended to subnormal, you can make up your mind that the case, if operated on, will die in twelve or twenty-four hours thereafter.

---

### RHEUMATIC PHARYNGITIS.

M. Braislin recommends:

| | | |
|---|---|---|
| ℞ | Acid salicylici | gr. xv. |
| | Ferri pyrophosphat | gr. iv. |
| | Sodii phosphat | gr. $\frac{3}{4}$. |
| | Aquæ | f ℥ ss. |

M. Sig.—For one dose. Repeat every four hours.

—*Rev. Intern. de Rhinologue, etc.*

